# Influence of molar incisor hypomineralisation severity on dental hypersensitivity, anxiety/fear, and aesthetic self-perception: a cross-sectional study

**DOI:** 10.1590/1678-7757-2024-0159

**Published:** 2025-07-07

**Authors:** Bianca Caroline GOMES, Lana CARDOSO-SILVA, Beatriz Kelly Barros LOPES, Roberta Paula de Faria MELO, Isabella Silva CATANANTI, Alexandra Mussolino de QUEIROZ, Francisco Wanderley Garcia PAULA-SILVA, David John MANTON, Fabrício Kitazono de CARVALHO

**Affiliations:** 1 Universidade de São Paulo Faculdade de Odontologia de Ribeirão Preto Departamento de Odontopediatria Ribeirão Preto Brasil Universidade de São Paulo, Faculdade de Odontologia de Ribeirão Preto, Departamento de Odontopediatria, Ribeirão Preto, Brasil.; 2 University of Groningen University Medical Centre Groningen Department of Cariology Groningen Netherlands University of Groningen, University Medical Centre Groningen, Centre for Dentistry and Oral Hygiene, Department of Cariology, Groningen, The Netherlands.

**Keywords:** Molar incisor hypomineralisation, Dental hypersensitivity, Dental anxiety, Children

## Abstract

Molar-incisor hypomineralisation (MIH) significantly impacts children's quality of life due to dental hypersensitivity, anxiety, and aesthetic concerns. Few studies have investigated these factors within the same sample, comparing varying levels of MIH severity to MIH-free controls.

**Objectives:**

This study evaluated dental hypersensitivity, anxiety, and aesthetic self-perception in children with mild MIH, severe MIH, and controls, and explored correlations between the studied variables.

**Methodology:**

A total of 605 students from a single school were evaluated: 110 children with MIH and 214 controls matched by age and sex. MIH severity was clinically assessed using the Ghanim criteria. Hypersensitivity was measured with the visual analogue scale (VAS) and the Schiff Cold Air Sensitivity Scale (SCASS). Anxiety and aesthetic concerns were assessed using the CFSS-DS and CQATA questionnaires, respectively.

**Results:**

Overall, 78 children had mild MIH and 32 had severe MIH. Median for dental hypersensitivity (p<0.001) and aesthetic self-perception (p=0.002) were significantly higher in the severe MIH group compared to both the control and mild MIH groups. No differences were found for anxiety. Elevated VAS-measured dental hypersensitivity levels and impaired self-perceived aesthetics were significantly correlated with greater MIH severity (p<0.05). Spearman's correlation analysis further revealed significant positive correlations between anxiety/fear and VAS scores (p=0.023, r=0.239), between anxiety/fear and aesthetic self-perception scores (p=0.007, r=0.282), and between dental appearance classification and VAS hypersensitivity scores (p=0.035, r=0.222). In contrast, SCASS hypersensitivity scores did not significantly correlate with either anxiety/fear or dental appearance classification (p>0.05).

**Conclusions:**

Children with severe MIH showed higher dental hypersensitivity and greater perceived aesthetic impairment than children with mild MIH or without MIH.

## Graphical abstract

**Figure:**
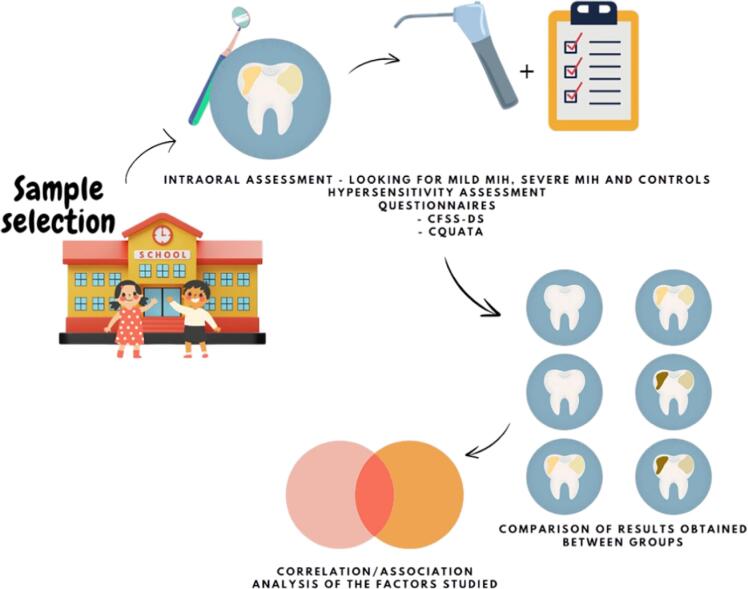

## Introduction

Molar incisor hypomineralisation (MIH), a qualitative enamel defect, is characterized by demarcated opacities ranging from white-creamy to yellowish-brown. MIH affects one or more permanent first molars and the incisors may or may not be involved.^1,2^

MIH may also be associated with dental hypersensitivity, which can have impact on the daily lives and quality-of-life of the affected individuals. Several studies have been conducted to assess the presence and levels of hypersensitivity in cases of MIH, whether spontaneous or triggered by thermal, osmotic, and mechanical stimuli.^3–6^ However, the correlation between dental hypersensitivity intensity and the severity of the defect remains relatively unexplored.

A prior systematic review assessed anxiety related to MIH in clinical studies and found that currently it is not possible to determine if children with MIH had increased fear during dental treatment.^7^ However, the importance of research correlating fear with the severity of MIH has been emphasized because more severe cases may demonstrate a need for fear and anxiety management.^8^ Vicioni-Marques and colleagues reported no significant association between anxiety/fear and hypersensitivity in MIH; however, there was no control for children without MIH.^6^

Assessments of the repercussions of the clinical characteristics of MIH on the patient's life are important and gaining momentum in scientific research. For instance, it has been suggested that self-perceived impaired aesthetics can directly interfere with social interaction or even disrupt an individual's daily routines.^9,10^ The aesthetic self-perception of parents and children related to MIH has been evaluated by quantifying aesthetic satisfaction and dental concerns.^11,12^ These studies did not explore associations between aesthetic self-perception and other factors, such as anxiety or dental hypersensitivity, but they suggested that the clinical characteristics of demarcated opacities may lead to a negative self-image, especially in cases of severe MIH. Other authors suggested there may be an influence of MIH severity on greater involvement of other teeth, meaning that more severe MIH in the permanent first molars leads to a greater impact on the anterior teeth.^2,13^ There is no clarity whether there is a relationship between increased hypersensitivity in severe cases of MIH, anxiety, and a child's aesthetic self-perception.

This study aimed to evaluate dental hypersensitivity, anxiety, and self-perception of dental aesthetics in children with mild MIH, severe MIH, and a control group of children without enamel defects. The study also aimed to establish whether correlations between the evaluated parameters.

## Methodology

### Study data

This cross-sectional observational study was conducted in a municipal school located in the West Zone of Ribeirão Preto, São Paulo, Brazil from August to December 2022. The city currently has a population of approximately 698,000 people including 81,000 individuals aged under 14 years: 41,000 males and 40,000 females.^14^

Sample size calculations were performed for all outcomes, in which the final sample was determined by the largest requirement. For dental anxiety, using the adjusted proportion estimation formula for a finite population — with a prevalence rate of 35%, a 95% confidence interval, a 5% margin of error, and a design effect of 1.7 — a minimum sample of 594 children was needed. For dental hypersensitivity, based on data from Raposo, et al.^5^ (2019) and an estimated prevalence of 34.7% (versus a baseline prevalence of 15%), 73 children per group were required to achieve 80% statistical power at a 5% significance level. A total of 109 children were needed for self-perceived aesthetics, using findings from Fragelli, et al.^12^ (2021) — which associated MIH in anterior teeth with self-perception of aesthetics, assuming a standard deviation of 1.31 points and a detectable difference of 0.5 points under the same power and significance criteria. Therefore, the final sample size was based on the dental anxiety calculation, as it required the largest number of participants.

A public school in a low socioeconomic region that met the required sample size within the study's age range was selected resulting in a convenience sample. This project was approved by the Research Ethics Committee of the Ribeirão Preto School of Dentistry, University of São Paulo. The Strengthening the Reporting of Observational Studies in Epidemiology (STROBE) guidelines were followed to report this cross-sectional study^15^. Participation in the study was voluntary, and consent and assent forms were obtained from the participants and their guardians.

### Examiner calibration

Two examiners (B.C.G and L.C.S) were trained and calibrated by a reference examiner experienced in MIH research. Calibration was performed following the criteria outlined by Ghanim, et al.^16^ (2015), and its protocol can be found in Vieira, et al.^17^ (2023) and is summarized below.

The examiners received comprehensive explanations regarding concepts, clinical characteristics, and utilised indices. After the theoretical discussions, the examiner conducted two evaluative/educational sessions for practical calibration using approximately 30 images from the department's own archives. These images contained teeth in different clinical conditions including healthy teeth, i.e., teeth without any defect in dental development, hypomineralisation, fluorosis, hypoplasia, and amelogenesis imperfecta.

Dental enamel clinical calibration was further refined via the evaluation of 20 children undergoing treatment. The inter- and intra-examiner agreement achieved a Kappa coefficient > 0.8.

### Sample selection

All children at the school were assessed at baseline; 605 children were enrolled at that time, and they were six to 12 years old and of both sexes. They also received health education guidance and supervised brushing. An intraoral examination was performed by the B.C.G examiner in the schoolyard under natural light conditions with the patient lying down and assisted by a mirror, wooden spatula, and head-mounted LED flashlight (NOLL-AMATOOLS – model 3510003, Piracicaba-SP, Brazil). Full personal protective equipment (PPE) including a gown, cap, mask, and gloves were worn during the examination.

**Figure 1:**
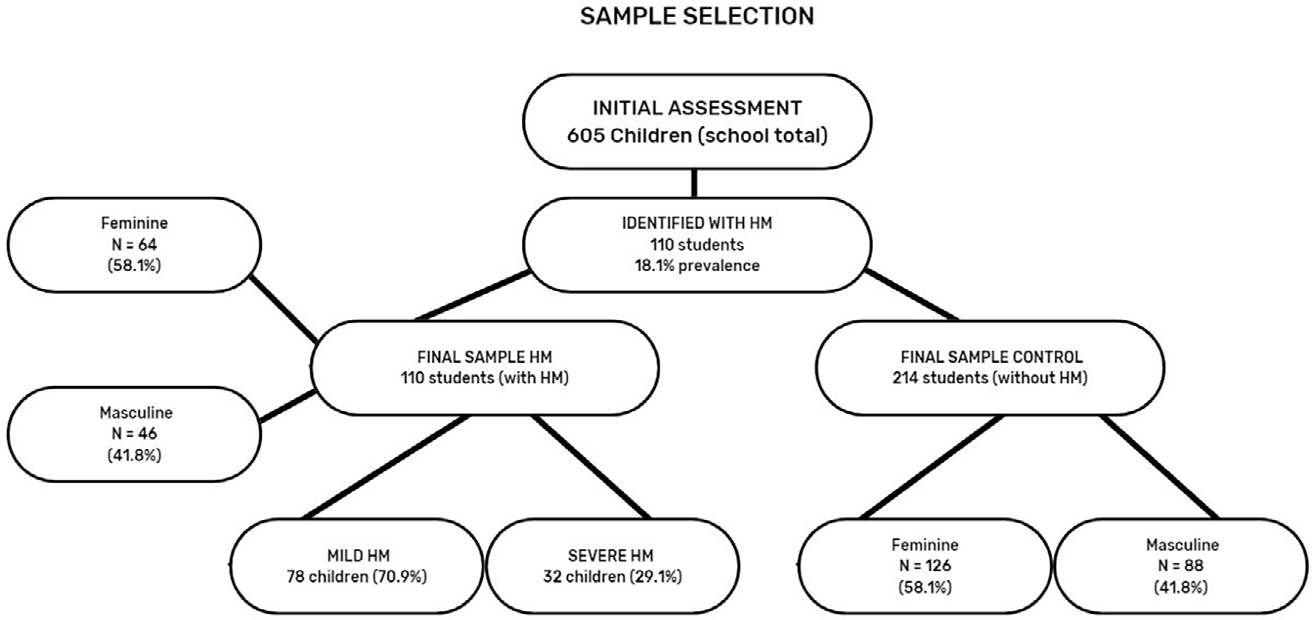
Study sample flowchart

In the second phase, children with MIH (n=110) were included in the study and subsequently divided into two groups based on its severity: mild MIH (n=78) (children with one or more first permanent molars (FPM) showing demarcated white, yellow, or brown opacities) and severe MIH (n=32) (children with at least one FPM showing fractures exposing enamel and/or dentin). For each child with MIH, two children without any enamel defects due to MIH were included in the control group (children without MIH, n=214) (Figure 1). The participants in the control group could have carious lesions (ICDAS 4 at most), and six children from the control group were excluded due to absence on the day of evaluation. Considering the presence or not of MIH involving the anterior teeth, the MIH group (n = 110) was composed of 48 children with MIH in both posterior and anterior teeth (36 with mild MIH and 12 with severe MIH) and 62 children with MIH only in the posterior teeth (42 with mild MIH and 20 with severe MIH).

Therefore, the inclusion criteria for the MIH group were the presence of at least one affected first permanent molar (FPM). Considerations for the inclusion of pairs in the control group included classroom similarity, sex, age similarity, and absence of enamel developmental defects. The exclusion criteria encompassed children with carious lesions (ICDAS ≥ 5), orthodontic appliances, or other dental developmental defects such as fluorosis, amelogenesis imperfecta, or dentinogenesis imperfecta.

### Dependent variables assessment

For descriptive analyses, the number of teeth and surfaces affected by MIH were compared among the groups, considering permanent first molars and incisors.

Dental hypersensitivity was assessed by two scales and an air jet stimulus was applied with a portable dental unit (Portable Dental Equipment – Dental Case, São Paulo, Brazil). The triple syringe was positioned 1 cm from the tooth, directed toward the tooth surface, and activated for three seconds. The child's dental hypersensitivity response was recorded by the VAS scale, ranging from zero (no dental hypersensitivity) to ten (unbearable dental hypersensitivity). The child's reaction to the stimulus was also observed and classified using the SCASS scale, with scores ranging from zero (no response) to three (withdraws and requests to stop immediately). The test was conducted by first familiarizing each child with the procedure, in which a test was performed on healthy teeth so that the child could experience the sensation of the air stimulus. The presence of hypersensitivity was considered when the child showed a positive response to the stimulus on either the VAS or SCASS scale.^18–20^ Thus, for analytical purposes, hypersensitivity data were treated as ordinal qualitative variables, categorizing pain as either present or absent based on at least one positive evaluation.

The Brazilian version of the Children's Fear Survey Schedule-Dental Subscale (CFSS-DS) was used to assess fear and anxiety levels.^21^ This questionnaire consists of 15 questions evaluating fear in various situations, and the responses were recorded using a 5 point Likert scale, enabling anxiety levels to range from 15 (no anxiety) to 75 (maximum anxiety)^5^.

The questionnaire used to assess self-perceived dental aesthetics consisted of seven questions adapted from the Child Perceptions Questionnaire About Tooth Appearance (CQATA)^22^ validated for Brazilian Portuguese.^23^ The questions were divided into two parts. In the first part, which we called "Aesthetics", the questions evaluate discomfort, worry, and difficulties in smiling over the past two months. Ratings ranged from "not at all," "very little," "a little," to "a lot," which corresponded to values of one to four, respectively. The child was considered dissatisfied or perceived impaired esthetics if the sum was six or more points. The "Dental Aesthetics Rating" questions addressed dental ratings such as dental alignment, teeth colour, and healthy appearance. Responses were also numerical and ranged from one to five, in which a child was considered aesthetically unsatisfied if the sum was ten or more.^12^ All questionnaires were applied by a second examiner (L.C.S) after the intra-oral examination without knowing which group the child was assigned to.

### Statistical analysis

Quantitative data were analysed for distribution using the Kolmogorov-Smirnov test, which indicated a non-normal distribution (p<0.05). Therefore, dependent variables were compared between groups using the non-parametric Kruskal-Wallis test, followed by Dwass-Steel-Critchlow-Fligner pairwise comparisons and the Mann-Whitney test for comparisons between MIH children with or without anterior teeth affected. Spearman's correlation test was used to assess correlations between variables and the number of teeth affected by MIH.

## Results

The mean ages were similar across the groups: 9.07 years for the control group, 8.96 years for the mild MIH group, and 9.31 years for the severe MIH group (p=0.59). Regarding the number of affected teeth, no statistically significant differences were found between the mild and severe MIH groups, whether molars or incisors. However, for the number of affected surfaces in general, paired comparisons revealed a significantly higher median for the severe MIH group compared to the mild MIH group (Table 1).

**Table 1:** Descriptive Data of the Sample for Each Severity Level: (1) Control; (2) Mild MIH; (3) Severe MIH Median (25^th^-75^th^).

Groups/Outcomes	Total number of teeth affected by MIH (PFM + Incisors)	Total number of PFM affected by MIH	Total number of incisors affected by	Total number of dental surfaces affected by
**(p value)**	(p<0.001)	(p<0.001)	MIH (p<0.001)	MIH (p<0.001)
**Control**				
Median	0.0^a^	0.0^a^	0.0^a^	0.0^a^
(25^th^-75^th^)	(0.0 – 0.0)	(0.0 – 0.0)	(0.0 – 0.0)	(0.0 – 0.0)
**Mild**				
Median	3.0^b^	2.0^b^	0.0^b^	4.0^b^
(25^th^-75^th^)	(2.0 – 4.0)	(1.0 – 3.0)	(0.0 – 1.0)	(3.0 – 6.0)
**Severe**				
Median	4.0^b^	2.5^b^	0.0^b^	5.5^c^
(25^th^-75^th^)	(3.0 – 5.25)	(1.0 – 4.0)	(0.0 – 1.2)	(4.7 - 9.0)

Different letters represent a statistically significant difference between the upper and lower rows of the same column (Kruskal-Wallis Test with pairwise comparison).

Table 2 shows comparative analyses of the dependent variables — dental hypersensitivity, anxiety, and aesthetic self-perception. The severe MIH group showed significantly higher dental hypersensitivity compared to both the mild MIH and control groups (p<0.001). The percentages of children reporting dental hypersensitivity were 19% in the control group, 14.3% in the mild MIH group, and 59.4% in the severe MIH group. Median anxiety scores were similar across the three groups, ranging from 25.0 to 26.0 points (considering a cutoff of ≥ 38 points for anxiety/fear), with no significant differences observed (p=0.82). In the outcome of aesthetic self-perception, there was a statistically significant difference between the severe MIH group and the other groups, with higher scores observed in both aesthetic parameters (aesthetics and dental aesthetics rating). The cutoff points for these questionnaires were ≥ 6 points for the first and ≥ 10 points for the second. When the variables of anxiety and self-perception of dental aesthetics were compared between the groups of children with MIH affecting or not the anterior teeth, no statistically significant differences were found (Table 3).

**Table 2:** Main outcome data for groups. Kruskal-WallisTest with pairwise comparison.

Groups/Outcomes	Dental Hypersensitivity Presence	Aesthetics	Dental Esthetics Rating
**(p value)**	(p<0.001)	(p=0.015)	(p=0.002)
**Control**	19%		
Median	1.0^a^	5.0^a^	11.0^a^
(25^th^-75^th^)	(1.0 – 1.0)	(3.0 – 7.0)	(9.0 – 12.0)
**Mild**	14,3%		
Median	1.0^a^	5.0^a^	10.0^a^
(25^th^-75^th^)	(1.0 – 1.0)	(3.0 – 8.0)	(8.0 – 14.0)
**Severe**	59,4%		
Median	2.0^b^	7.0^b^	12.0^b^
(25^th^-75^th^)	(1.0 – 2.0)	(5.0 – 9.0)	(11.0 – 15.0)

(Different letters represent a statistically significant difference between the upper and lower rows of the same column).

**Table 3:** Dependent variable data for MIH children with or without affected anterior teeth.

Groups/Variables	Number of teeth affected by MIH	Anxiety	Aesthetics	Dental Esthetics Rating
**(p value)**	(p<0.001)	(p=0.397)	(p=0.274)	(p=0.324)
**Control Group**				
Median	0^b^	26^a^	5^a^	11^a^
(25^th^-75^th^)	(0.0 - 0.0)	(21.0 - 33.0)	(3.0 - 7.0)	(9.0 - 12.0)
**MIH with Anterior Affected**				
Median	4^a^	27^a^	6^a^	12^a^
(25^th^-75^th^)	(3.0 – 6.0)	(21.0 – 33.5)	(4.7 – 8.2)	(9.7 – 14.0)
**MIH posterior teeth only**				
Median	3^a^	25^a^	5^a^	11^a^
(25^th^-75^th^)	(2.0 – 4.0)	(21.0 – 31.0)	(3.0 – 8.0)	(9.0 – 14.0)

Kruskal-Wallis test by Dwass-Steel-Critchlow-Fligner pairwise comparisons (Different letters represent a statistically significant difference between the upper and lower rows of the same column).

The Spearman correlation test revealed positive correlations between anxiety/fear and VAS scores (p=0.023, r=0.239), anxiety/fear and Aesthetics rating (p=0.007, r=0.282), as well as between the Aesthetics rating and hypersensitivity (p=0.035, r=0.222). However, no statistically significant correlation was found between SCASS hypersensitivity aspects and the level of anxiety/fear, or between anxiety/fear and the classification of Aesthetics and dental aesthetics rating (p>0.05).

## Discussion

This study showed that children with severe MIH had the most unfavourable outcomes in terms of hypersensitivity and aesthetic self-perception. These findings suggest a negative synergistic impact of MIH that increases with defect severity. A recent study by Azfal, et al.^23^ (2024) also demonstrated that children with severe MIH had a higher total number of affected teeth, more anterior teeth with MIH, and a greater prevalence of dental hypersensitivity. These findings corroborate this study and suggest that in cases of severe MIH, increased hypersensitivity, poorer aesthetic self-perception are observed in affected children.

The hypersensitivity associated with MIH, although not yet fully understood, may be linked to the high porosity of the affected enamel, which facilitates the penetration of bacteria into the exposed dentinal tubules, leading to a subclinical condition of pulp inflammation.^4^ Furthermore, even in cases of opacities without structural loss, sensitivity can occur in response to mild external stimuli, potentially progressing to spontaneous hypersensitivity.^24^ The prevalence of dental hypersensitivity in our study was 14.3% for mild MIH and 59.4% for severe MIH, whereas Raposo, et al.^5^ (2019) showed the reported prevalence was 29.7% for mild MIH and 51.6% for severe MIH. This slight difference in the data is likely because Raposo, et al.^5^ (2019) used a different classification system for the severity of hypomineralisation, which included a third group, considering moderate MIH. However, this classification is no longer recommended, with current guidelines advocating only for the use of mild and severe MIH categories.^2^ The decision to analyse dental hypersensitivity as a dichotomous variable was driven by the challenges inherent in paediatric pain assessment. While a zero to ten quantitative scale can provide nuanced information on pain intensity, many children have difficulty differentiating subtle variations, which may lead to inaccuracies. Dichotomizing the variable simplifies interpretation and minimizes bias related to self-report variability, aligning with previous research, such as Raposo, et al.^5^ (2019) and Azfal, et al.^23^ (2024), which employed a similar method to assess hypersensitivity in children with MIH. Another important point is that due to difficulties in finding children with healthy teeth for the control group sample, children with caries lesions (maximum ICDAS 4) were admitted. This factor may have influenced the results, in which higher hypersensitivity is found in the severe MIH group compared to the other groups, but not when comparing the mild MIH and control groups. This may occur because the children in the control group showed hypersensitivity related to other conditions.

When hypersensitivity is present, the patient may experience fear and/or anxiety regarding dental treatment. Considering a cutoff point of ≥ 38 points for anxiety/fear, our sample did not demonstrate a consistent pattern of anxiety related to dental care with a mean score of 26 points, which is similar across the three evaluated groups and is comparable to Laureano, et al.^25^ (2020) who reported a mean score of 29.9. Other studies have failed to show a relationship between increased dental anxiety or fear in MIH including those that used the CFSS-DS questionnaire.^6,7,26–29^ We must consider that the questionnaire is not specific to the particularities of MIH or to questions regarding dental anxiety/fear itself, so new studies are needed with new questionnaires that include these questions in a more specific way.

Regarding anxiety/fear in our analyses, statistically significant differences were not found in comparisons between the MIH groups and the control group. Jälevik and Klingberg^8^ (2002) reported more behavioural management issues and higher levels of anxiety and fear related to dental treatment in children with severe MIH compared to the control group. However, unlike this study, which observationally analysed these aspects, Jälevik and Klingberg^8^ (2002) assessed them in the context of dental treatments, which may have influenced the participants’ perceptions to some extent. We speculate that the school environment may provide a calmer setting for children compared to the dental environment, which could have influenced our results. Therefore, further studies are recommended to investigate children with severe MIH in different settings to explore this finding in greater depth. Children with severe MIH tend to have higher levels of hypersensitivity. Furthermore, Jalevik et al.^7^ (2002) show that children with severe MIH have greater behaviour management issues. An interesting observation in our study is that there was a correlation, although weak, between anxiety levels and the VAS hypersensitivity measures. Therefore, the association between behaviour management issues, anxiety, and higher levels of hypersensitivity should still be investigated more deeply in future studies.

Regarding aesthetic perceptions the group of children with severe MIH showed significantly higher scores for poor aesthetic self-perception, with no statistical difference observed in this dependent variable between the control and mild MIH groups. In the first questionnaire, only the severe MIH group reached the threshold score (six points) considered indicative of aesthetic dissatisfaction. Interestingly, in the second questionnaire, despite the statistical difference between the groups and severe MIH, all of them had scores greater than ten, the minimum score corresponding to an unsatisfactory classification of dental aesthetics. This may have been due to factors not considered in the exclusion criteria, such as orthodontic issues, poor dental alignment, and restorations with defects in the anterior teeth of the children in the control group. Nonetheless, the severe MIH group still had higher scores for unsatisfactory appearance classification, showing that the results were slightly different from a previous study, which indicates that children with MIH have worse self-perception of dental aesthetics and more negative perceptions of dental health.^12^ Curiously, in the comparative analyses between the MIH groups, statistically significant differences were found only for the number of affected surfaces, being greater in the severe group, but not in the analyses of the number of anterior and posterior teeth. Additionally, it was not possible to use the same three-group division for the analysis of anterior teeth affected by MIH, as the occurrence of post-eruptive breakdown and/or carious lesions or atypical restorations in these teeth is rarer.

Fragelli, et al.^12^ (2021), showed a greater aesthetic impact in children with MIH affecting the anterior teeth, which was not replicated in our study. This discrepancy may be due to the relatively low number of anterior teeth affected in our sample, with a mean of only one affected incisor per child across both the mild and severe MIH groups. Even when considering only children with anterior teeth affected, the median number of affected incisors was still low (2). Future studies focusing specifically on children with more extensive anterior tooth involvement may provide clearer insights into the aesthetic impact of MIH. Note that the measure of aesthetic self-perception used in our study is somewhat subjective and may vary depending on the child's overall perception of their appearance and well-being at the time of assessment. Additionally, our study found a weak positive correlation between anxiety/fear and aesthetic self-perception scores, which aligns with previous research showing that individuals with higher levels of anxiety and/or fear towards dental treatment tend to have greater dissatisfaction with their orofacial appearance.^30^

There was a weak correlation between hypersensitivity and aesthetic perception, suggesting the painful sensation could draw attention to teeth that previously did not cause concern, which could lead to a compromised aesthetic perception due to the symptoms. Note that other studies have identified self-esteem issues related to increased pain and a reduction in quality of life due to other oral health problems.^31,32^ The potential association between severe MIH, aesthetic self-perception, and psychological impacts, such as self-esteem and sense of coherence, may be worth investigating in future studies.

We observed some limitations, such as the fact that the questionnaire used was not specific to individuals with MIH. Additionally, the age of the children and the fact that the SCASS test appeared to be somewhat challenging for them should be considered, as pain is a subjective experience. Moreover, some children had difficulty interpreting certain terms used in the CFSS-DS and CQATA questionnaires, then some words were orally modified and replaced by synonyms to ensure better understanding. An observational study provides a snapshot of a specific moment, therefore, new studies are needed to include assessments at different stages and time points. Our findings highlight how severe MIH can significantly affect those who suffer from it, so it is crucial to disseminate information and pay attention to cases of MIH to prevent these defects from severely impacting the child. Future studies focusing on the development of specific questionnaires for MIH are recommended, addressing issues such as anxiety, aesthetic self-perception, and their impact on the quality of life of children with MIH.

## Conclusions

Children with severe MIH had higher dental hypersensitivity and greater perceived aesthetic impairment than children with mild MIH or without MIH.

## Data availability statement

The datasets generated during and/or analyzed during the current study are available from the corresponding author on reasonable request.
